# Effects of propofol on inflammatory response and activation of p38 MAPK signaling pathway in rats with ventilator-induced lung injury

**DOI:** 10.1590/ACB361004

**Published:** 2021-11-22

**Authors:** Jiandong Deng, Mingqin Xiong, Caiping Liao, Tao Xiang

**Affiliations:** 1 Bachelor. Department of Anesthesiology - The First Hospital of Changsha, China.; 2 Bachelor - Department of General Surgery – The First Hospital of Changsha, China.; 3 Master. Department of Anesthesiology - The First Hospital of Changsha, China.

**Keywords:** Propofol, Ventilators, Mechanical, Lung Injury, Inflammatory Response, Innate, p38 MAPK, Rats

## Abstract

**Purpose:**

To investigate the effects of propofol on inflammatory response and activation of p38 mitogen-activated protein kinase (MAPK) signaling pathway in rats with ventilator-associated lung injury (VALI).

**Methods:**

Thirty-six Sprague Dawley (SD) rats were divided into control, VALI and VALI+propofol groups. The VALI group received the mechanical ventilation for 2 h. The VALI+propofol group received the mechanical ventilation for 2 h, which was accompanied by intravenous injection of propofol with dose of 8 mg·kg^-1^·h^-1^. At the end, the mean arterial pressure (MAP) and blood gas indexes were measured, and the lung wet/dry mass ratio (W/D) and biochemical indexes of lung tissue and bronchoalveolar lavage fluid (BALF) were determined.

**Results:**

Compared with VALI group, in VALI+propofol group the blood pH, partial pressure of oxygen, partial pressure of carbon dioxide and MAP were increased, the lung W/D, lung tissue myeloperoxidase activity and total protein concentration, white blood cell count, and tumor necrosis factor α, interleukin 1β and interleukin 6 levels in BALF were decreased, and the p-p38 MAPK protein expression level and phosphorylated p38 MAPK (p-p38 MAPK)/p38 MAPK ratio were decreased.

**Conclusions:**

Propofol treatment may alleviate the VALI in rats by reducing the inflammatory response and inhibiting the activation of p38 MAPK signaling pathway.

## Introduction

In recent years, the clinical application of ventilator is increasing, but the lung injury caused by improper use of ventilator or its long application time has gradually attracted the attention of clinicians^1^. If the ventilator-associated lung injury (VALI) cannot be treated in time, it will seriously endanger the respiratory function of patients, and even lead to death. At present, it is believed that the variation of proinflammatory mediators, changes in cell conformation, remodeling of cytoskeleton, activation of signal transduction pathway and apoptosis of pulmonary epithelial cells play important roles in VALI^2^^,^ 4.

In clinic, VALI is mainly treated by carbon dioxide inhalation, anti-inflammatory treatment, adrenoceptor drug therapy and renin-angiotensin-aldosterone system drug therapy, but there is no drug to specifically improve the prognosis of VALI. Propofol is an alkyl acid short-acting intravenous sedative which is currently used for the induction and maintenance of anesthesia, especially for minor surgery, outpatient biopsy and sedation of patients in critical care unit^5^^,^ 6. It is found that propofol can alleviate the vomiting, immune response, anxiety, and pain, besides protecting the nervous system^7^. Propofol is also a commonly used drug for sedation during mechanical ventilation. It has anti-inflammatory properties, can reduce the lung injury and improve the oxygenation during mechanical ventilation, but the mechanism is unclear^8^^,^ 9.

It is found that the p38 mitogen-activated protein kinase (MAPK) signaling pathway is closely related to the lung injury^10^^,^ 12. This study was designed to investigate the effects of propofol on inflammatory response and activation of p38 MAPK signaling pathway in rats with VALI.

## Methods

This study was approved by the ethics committee of the First Hospital of Changsha. All animal procedures were in accordance with the Guide for the Care and Use of Laboratory Animals by the National Institutes of Health.

### Animals and grouping

Thirty-six specific-pathogen-free (SPF)-grade male Sprague Dawley rats (250-300g; Shanghai Slake Experimental Animal Co.) were maintained under condition of 12-h/12-h light/dark cycle, temperature of 22±2°C and humidity of 50±5%. After one week of adaptive feeding, the rats were randomly divided into control, VALI and VALI+propofol groups (12 animals in each group).

### Establishment of VALI model and treatment

In VALI and VALI+propofol groups, the rats were anesthetized with 13.3% urethane combined with 0.5% chloraldose, followed by fixing. The trachea was cut open. A 16-gauge venous indwelling catheter was inserted into the trachea and connected to a small animal ventilator for mechanical ventilation. The tidal volume was set as 30mL/kg, and the ventilation time was 2h. In addition, in VALI+propofol group, during the mechanical ventilation, propofol was intravenously injected, with dose of 8 mg·kg^-1^·h^-1^. In control group, the rats received the spontaneous breathing, without mechanical ventilation.

### Mean arterial pressure monitoring and blood gas analysis

At the end of mechanical ventilation, the mean arterial pressure (MAP) of rats was monitored. In addition, 0.3 mL of arterial blood was collected for blood gas analysis. The blood pH, partial pressure of oxygen (PaO_2_), and partial pressure of carbon dioxide (PaCO_2_) were measured.

### Determination of lung wet/dry mass ratio

The rats were sacrificed. The right middle lung tissue was taken. The blood and water on the surface were sucked dry with absorbent paper. The lung tissue was weighed to obtain the wet mass (mg). Then, the lung tissue was dried in 80°C drying oven to constant weight to obtain the dry mass (mg). The lung wet/dry mass ratio (W/D) was obtained.

### Determination of biochemical indexes of bronchoalveolar lavage fluid

The lavage of left lung was performed using normal saline. The bronchoalveolar lavage fluid (BALF) was obtained, followed by centrifuging at 1,000 r/min for 10 min. The precipitate was diluted with phosphate buffered saline. After Wright’s staining, the white blood cells were counted under light microscope. The total protein concentration in BALF was determined by Coomassie brilliant blue staining. The tumor necrosis factor α (TNF-α), interleukin 1β (IL-1β) and interleukin 6 (IL-6) were detected by double-antibody sandwich indirect enzyme-linked immunosorbent assay. All operations were carried out in strict accordance with the requirements of the kit instructions.

### Determination of biochemical indexes of lung tissue

The left lung tissue was taken and homogenized. The protein was extracted using radio-immunoprecipitation assay (RIPA) buffer, and the protein concentration was determined by Coomassie brilliant blue method. The myeloperoxidase activity in lung tissue was measured using the specific kit. The expressions levels of p38 MAPK and phosphorylated p38 MAPK (p-p38 MAPK) proteins were determined using western blot assays. The procedures were in accordance with the instructions of kits.

### Statistical analysis

Data were presented as mean±standard deviation and analyzed using Statistical Package for the Social Sciences (SPSS) 18.0 software. The comparisons among three groups were performed using single-factor analysis of variance test with post-hoc Student-Newman-Keuls (SNK) test. P < 0.05 and P < 0.01 were considered significant and highly significant, respectively.

## Results

### Comparison of blood pH, PaO_2_, PaCO_2_ and MAP among three groups 

At the end of mechanical ventilation, the blood pH, PaO_2_, PaCO_2_ and MAP had significant difference in three groups, respectively (P < 0.001). Compared with control group, each index in VALI and VALI+propofol groups was significantly decreased, respectively (P < 0.01). Compared with VALI group, each index in VALI+propofol group was significantly increased (P < 0.01) (Table 1).

**Table 1:** Comparison of blood pH, PaO_2_, PaCO_2_ and MAP among three groups.

Group	pH	PaO_2_ (mmHg)	PaCO_2_ (mmHg)	MAP (mmHg)
Control	7.40±0.04	99.30±14.72	40.17±6.35	123.06±5.40
VALI	7.16±0.05 a	65.06±10.20 a	69.54±8.19 a	89.12±6.19 a
VALI+propofol	7.30±0.04 a b	78.28±7.06 a b	56.68±7.04 a b	103.32±7.04 a b
F	91.789	28.967	49.715	89.368
P	< 0.001	< 0.001	< 0.001	< 0.001

a P < 0.01 *vs*. control group;

b P < 0.01 *vs*. VALI group;

VALI: ventilator-associated lung injury; PaO_2_: partial pressure of oxygen; PaCO_2_, partial pressure of carbon dioxide; MAP, mean arterial pressure.

### Comparison of lung W/D and lung tissue myeloperoxidase activity among three groups

As shown in Table 2, at the end of mechanical ventilation, the lung W/D and lung tissue myeloperoxidase activity had significant difference in three groups (P < 0.001). Each index in VALI and VALI+propofol groups was significantly higher than that in control group (P < 0.01), and each index in VALI+propofol group was significantly lower than that in VALI group (P < 0.01).

**Table 2:** Comparison of lung W/D and lung tissue myeloperoxidase activity among three groups.

Group	Lung W/D	Myeloperoxidase (U/g)
Control	3.54±0.56	0.85±0.12
VALI	5.18±0.78 a	2.38±0.37 a
VALI+propofol	4.37±0.39 a b	1.43±0.19 a b
F	22.538	114.615
P	< 0.001	< 0.001

a P < 0.01 *vs*. control group;

b P < 0.01 *vs*. VALI group;

VALI: ventilator-associated lung injury; W/D: wet/dry mass ratio; F: statistics of single-factor analysis of variance test.

### Comparison of total protein concentration and white blood cell count in BALF among three groups

At the end of mechanical ventilation, there was significant difference of total protein concentration and white blood cell count in BALF among three groups (P < 0.001). Compared with control group, each index in VALI and VALI+propofol groups was significantly increased (P < 0.01). Compared with VALI group, each index in VALI+propofol group was significantly decreased (P < 0.01) (Table 3).

**Table 3:** Comparison of total protein concentration and white blood cell count in BALF among three groups.

Group	Total protein concentration (g/L)	White blood cell count (1×10^7^ /L)
Control	0.41±0.06	39.92±5.06
VALI	1.48±0.18 a	121.38±19.37 a
VALI+propofol	0.72±0.12 a b	65.05±9.28 a b
F	216.500	128.650
P	< 0.001	< 0.001

a P < 0.01 *vs*. control group;

b P < 0.01 *vs*. VALI group;

VALI: ventilator-associated lung injury; F: statistics of single-factor analysis of variance test.

### Comparison of TNF-α, IL-1β and IL-6 levels in BALF among three groups

Table 4 shows that, at the end of mechanical ventilation, TNF-α, IL-1βand IL-6 levels in BALF had significant difference in three groups (P < 0.001). Each index in VALI and VALI+propofol groups was significantly higher than that in control group (P < 0.01), and each index in VALI+propofol group was significantly lower than that in VALI group (P < 0.01).

**Table 4:** Comparison of TNF-α, IL-1β and IL-6 levels in BALF among three groups.

Group	TNF-α (pg/mL)	IL-1β (pg/mL)	IL-6 (pg/mL)
Control	67.38±9.42	45.83±6.04	39.05±7.24
VALI	281.20±36.28 a	201.47±38.21 a	121.25±19.82 a
VALI+propofol	124.16±23.19 a b	89.84±15.33 a b	61.72±11.77 a b
F	227.321	133.833	111.150
P	< 0.001	< 0.001	< 0.001

a P < 0.01 *vs*. control group;

b P < 0.01 *vs*. VALI group;

VALI: ventilator-associated lung injury; TNF-α: tumor necrosis factor α; IL-1β: interleukin 1β; IL-6: interleukin 1β; F: statistics of single-factor analysis of variance test.

### Comparison of p38 MAPK and p-p38 MAPK protein expression levels in lung tissue among three groups

At the end of mechanical ventilation, there was no significant difference of p38 MAPK protein expression level in lung tissue among three groups (P > 0.05), with significant difference of p-p38 MAPK protein expression level among three groups (P < 0.001). Compared with control group, p-p38 MAPK protein expression level and p-p38 MAPK/p38 MAPK ratio in VALI and VALI+propofol groups were significantly increased (P < 0.01). Compared with VALI group, each index in VALI+propofol group was significantly decreased (P < 0.01) (Table 5).

**Table 5:** Comparison of p38 MAPK and p-p38 MAPK protein expression levels in lung tissue among three groups.

Group	p38 MAPK/β -actin	p-p38 MAPK/ β-actin	p-p38 MAPK/ p38 MAPK
Control	1.06±0.15	0.79±0.12	0.77±0.18
VALI	1.09±0.13	1.78±0.26 a	1.66±0.27 a
VALI+propofol	1.15±0.17	1.27±0.16 a b	1.11±0.04 a b
F	1.107	116.958	67.925
P	0.343	< 0.001	< 0.001

a P < 0.01 *vs*. control group;

b P < 0.01 *vs*. VALI group;

VALI: ventilator-associated lung injury; MAPK: mitogen-activated protein kinase; F: statistics of single-factor analysis of variance test.

## Discussion

In the emergency treatment or rescue of critically ill patients, the use of ventilator is the most common and effective for mechanical ventilation. Despite the protective ventilation strategy, VALI is still one of the high risk factors^13^^,^ 14. Therefore, it is an inevitable trend to explore measures to prevent VALI.

In this study, the protective effects of propofol on rats with VILI were investigated. Results showed that, after 2 h of mechanical ventilation, compared with control group, the blood pH, PaO_2_, PaCO_2_ and MAP in VALI and VALI+propofol groups were significantly decreased. Compared with VALI group, each index in VALI+propofol group was significantly increased. This indicates that the propofol treatment can improve the pulmonary function of rats during mechanical ventilation.

Lung W/D reflects the degree of pulmonary edema, and the total protein concentration in BALF reflects the pulmonary exudation. The lung tissue myeloperoxidase activity and white blood cell count reflect the white blood cell infiltration of lung tissue. In this study, at the end of mechanical ventilation, compared with control group, the lung W/D, lung tissue myeloperoxidase activity and total protein concentration and white blood cell count in BALF in VALI and VALI+propofol groups were significantly increased. Compared with VALI group, each index in VALI+propofol group was significantly decreased. It suggests that the VALI occurs in rats, and the propofol treatment can alleviate the VALI.

There are many abnormal changes of the immune system in VALI, which is closely related to the release of inflammatory factors and inflammatory response. At the same time, the inflammatory response will aggravate the VALI^15^. Previous study^16^ has found that there is abnormal inflammatory response in rat VALI model. After treatment by curcumin with anti-inflammatory properties, the VALI is alleviated, and the release of inflammatory factors and activation of neutrophils are decreased.

Another study^17^ has shown that pycnogenol extracted from pine bark can resist the VALI, and reduce the lung tissue TNF-α, IL-1β and IL-6 levels. In our study, at the end of mechanical ventilation, the TNF-α, IL-1β and IL-6 levels in BALF in VALI and VALI+propofol groups were significantly higher than those in control group, and each index in VALI+propofol group was significantly lower than that in VALI group. It confirms that the propofol treatment can decrease the inflammatory response, which is related to its alleviative effect on VALI in rats.

Studies have shown that the initiation of pulmonary inflammatory response is closely related to the activation of inflammatory signal transduction pathway. The p38 MAPK pathway plays a particularly important role in lung injury. This pathway is coupled with the mechanical stimulation sensor and is also related to the transcription and expression of a variety of inflammatory substances^18^^,^ 19. After p38 MAPK pathway is activated, the inactive threonine and tyrosine are phosphorylated to form p-p38 MAPK protein. p-p38 MAPK protein can up-regulate the expression of a variety of inflammatory factors^20^.

Results of this study showed that, at the end of mechanical ventilation, there was no significant difference of p38 MAPK protein expression level in lung tissue among the three groups. However, compared with control group, the p-p38 MAPK protein expression level and p-p38 MAPK/p38 MAPK ratio in VALI and VALI+propofol groups were significantly increased. Compared with VALI group, each index in VALI+propofol group was significantly decreased. It indicates that the p38 MAPK signaling pathway is activated in mechanical ventilation, and the propofol treatment can inhibit the activation of p38 MAPK signaling pathway, thus reducing the VALI.

## Conclusions

The propofol treatment can alleviate the VALI in rats. The mechanism may be associated with reducing the inflammatory response and inhibiting the activation of p38 MAPK signaling pathway. This study has provided a new theoretical basis for the application of propofol in VALI and a new clue for the prevention and treatment of VALI.

This study, however, has some limitations. Firstly, the sample size of animals is relatively small, which may affect the persuasiveness of the results. Secondly, there may be other mechanisms related to the protective effect of propofol on VALI, which have not been investigated here. These issues need to be solved in the follow-up research.
